# Case fatality and recurrent tuberculosis among patients managed in the private sector: A cohort study in Patna, India

**DOI:** 10.1371/journal.pone.0249225

**Published:** 2021-03-26

**Authors:** Sophie Huddart, Mugdha Singh, Nita Jha, Andrea Benedetti, Madhukar Pai

**Affiliations:** 1 Department of Epidemiology, Biostatistics and Occupational Health, McGill University, Montreal, Canada; 2 McGill International TB Centre, Montreal, Canada; 3 Faculty of Medicine, University of California–San Francisco, San Francisco, CA, United States of America; 4 World Health Partners, Patna, India; 5 Manipal McGill Centre for Infectious Diseases, Manipal Academy of Higher Education, Manipal, India; Keele University, UNITED KINGDOM

## Abstract

**Background:**

A key component of the WHO End TB Strategy is quality of care, for which case fatality is a critical marker. Half of India’s nearly 3 million TB patients are treated in the highly unregulated private sector, yet little is known about the outcomes of these patients. Using a retrospective cohort design, we estimated the case fatality ratio (CFR) and rate of recurrent TB among patients managed in the private healthcare sector in Patna, India.

**Methods:**

World Health Partners’ Private Provider Interface Agencies (PPIA) pilot project in Patna has treated 89,906 private sector TB patients since 2013. A random sample of 4,000 patients treated from 2014 to 2016 were surveyed in 2018 for case fatality and recurrent TB. CFR is defined as the proportion of patients who die during the period of interest. Treatment CFRs, post-treatment CFRs and rates of recurrent TB were estimated. Predictors for fatality and recurrence were identified using Cox proportional hazards modelling. Survey non-response was adjusted for using inverse probability selection weighting.

**Results:**

The survey response rate was 56.0%. The weighted average follow-up times were 8.7 months in the treatment phase and 26.4 months in the post-treatment phase. Unobserved patients were more likely to have less than one month of treatment adherence (32.0% vs. 13.5%) and were more likely to live in rural Patna (21.9% vs. 15.0%). The adjusted treatment phase CFR was 7.27% (5.97%, 8.49%) and at 24 months post-treatment was 3.32% (2.36%, 4.42%). The adjusted 24 month post-treatment phase recurrent TB rate was 3.56% (2.54%, 4.79%).

**Conclusions:**

Our cohort study provides critical estimates of TB patient outcomes in the Indian private sector, and accounts for selection bias. Patients in the private sector in Patna experienced a moderate treatment CFR but rates of recurrent TB and post-treatment fatality were low.

## Introduction

India has the largest tuberculosis (TB) epidemic in the world, accounting for 27% of cases and 31% of global deaths [1]. In line with the WHO End TB strategy [2], the Indian national tuberculosis program has paid increasing attention to the quality of TB care in the country, especially in the largely unregulated private sector. However, there is insufficient data available in the currently available literature to form a reliable estimate of a key measure of the quality of care, the case fatality ratio, for privately treated TB patients in India [3]. Private healthcare dominates the Indian medical sector and it’s engagement is critical for ending TB in the country.

### Private sector TB care in India and private provider interface agencies

Half of all Indian TB patients are treated in the private sector where quality of care is generally low [4, 5]. Unlike in the public sector, there is no systematic monitoring of privately treated TB patient and very few studies have addressed case fatality among Indian private sector TB patients [3]. One solution for improved quality of care in the private sector lies in Private Provider Interface Agencies (PPIAs) which have been successfully implemented in multiple regions in India [6]. Early evidence suggests that PPIAs have been effective in increasing TB case notifications, increasing rates of TB testing, and improving treatment completion rates [7, 8].

PPIAs recognize that private physicians have little incentive to refer their patients to the public sector as this means lost income. Additionally, patients may prefer to receive treatment with their family physician, despite the freely available care in public facilities, to avoid over-crowded conditions and impersonal treatment. PPIAs provide a suite of interventions to improve patients’ care, including physician training on TB, free TB diagnostics, medicines, and treatment monitoring, in a manner that aligns with private physician incentives to retain their patients [9].

### Measuring quality of TB care

Two critical measures of quality of TB care are the case fatality ratio (CFR) and the rate of recurrent TB after treatment completion. At the country level, CFRs are estimated as the “number of TB deaths divided by the estimated number of incident cases in the same years” and is expressed as a percentage [1]. In cohort studies, the CFR can be exactly calculated because the number of incident cases is fixed by design. The End TB strategy calls for a global TB CFR below 6.5% with an ideal CFR below 5%. Elevated CFRs suggest failures in the patient pathway of care such as treatment delay, diagnostics delay, incorrect treatment regimen, unaddressed comorbid conditions, or poor treatment adherence.

A TB recurrence is defined as an instance when a patient who had completed treatment is later diagnosed with another TB episode [10]. TB recurrence can occur under two scenarios: 1) when the initial treatment has failed and the patient has a relapse with the same TB strain or 2) when a patient is successfully cured only to be later re-infected. Without molecular epidemiology studies, it is difficult to separate failure-to-cure relapse from true re-infections, though both are markers of failures in TB care.

Failure to cure is due to either ineffective treatment or loss to follow-up during treatment. Re-infections indicate that patients were cured but that the medical or social conditions like malnutrition, HIV co-infection, and overcrowding that led to the first TB infection have not been addressed. In India, there is currently no routine, long-term monitoring of TB patients for recurrence but previous studies have estimated the recurrence rate as high as 10% [11] with most recurrent episodes occurring within 6 months of treatment cessation [12].

### Loss to follow-up and selection bias

A major methodological concern when estimating patient outcomes is patient loss to follow-up [13]. Patients who are lost to follow-up may systematically differ from those retained in follow-up because they were sicker, more transient, received less social support, etc. Excluding these patients from analyses can induce selection bias and biased estimates. As we have previously noted, the Indian TB literature has underutilized modern epidemiological corrections for selection bias induced by loss to follow-up [3].

Inverse probability selection weighting (IPSW) [14] is a causal inference based method which reweights observed patients to represent the full cohort, including unobserved patients. This method takes advantage of baseline data available on the cohort and provides straightforward-to-interpret marginal rather than conditional estimates, as compared to older epidemiological techniques such as regression-based corrections [13].

Our earlier systematic review on this topic [3] estimated a treatment CFR among all Indian TB patients of 5.16% (4.20%, 6.34%), but too few high-quality studies were available concerning case fatality among TB patients managed in the private health sector. To explore quality of care and patient outcomes in the Indian private sector, we conducted a retrospective cohort survey of 4,000 World Health Partner (WHP) PPIA-treated TB patients in Patna, India. From this cohort survey we estimated treatment phase and post-treatment phase CFRs as well as the rate of recurrent TB up to two years post-treatment. Additionally, we assessed patient demographics associated with the risk of fatality and recurrence using survival modelling. Loss-to-follow up was corrected for using IPSW.

## Methods

### Parent project

In 2013, WHP established a PPIA in Patna, Bihar, India, as part of the Universal Access to TB Care (**UATBC**) initiative, in collaboration with the Bihar state TB program and the National TB program. The Indian national TB program reports a 2% treatment case fatality ratio among TB patients in Bihar [15], but this rate is not adjusted for patients lost to follow-up. Among Bihar patients notified to the national TB program, 8.7% are retreatment cases [15]. Since its inception, the WHP PPIA has treated 89,906 TB patients and continues to enroll patients. This program is not a research activity–it is a service delivery program providing quality TB services to private sector patients in the city with the engagement of the local government and various partners. Private sector physicians recruited to the program are trained on the diagnosis and treatment of TB. Patients enrolled by their physician with the PPIA are provided with vouchers for free chest X-rays, molecular diagnostics and treatment. A WHP call center provides biweekly treatment adherence monitoring calls and counselling. Patients who are not reachable by phone or who require additional counselling are visited at home by field officers. Patients diagnosed with drug-resistant TB are referred to the public sector.

At enrollment, patient contact information, date of enrollment, age and sex are recorded. The PPIA database also captures whether patients have pulmonary (PTB) or extrapulmonary TB (EPTB), whether the patient was a new or re-treatment case, and patient reported adherence to treatment.

### Patient sampling and survey

A random sample of 4,000 patients was drawn from all adult patients treated by the PPIA in July 2018 (n = 54,538). Surveys were conducted between July 2018 and April 2019 for a maximum possible follow-up time for patients of 5.5 years. Patients were contacted by up to three phone calls. If all phone calls were unanswered, a field officer visited the address recorded at PPIA enrollment. Patients or their next of kin who provided oral consent were administered a short survey asking if the patient had died and/or initiated another round of TB treatment and the date of either event. Patients or their next of kin were also asked if the patient had continued treatment after the 6–9 months provided by the PPIA. Survey responses were collected on paper forms before digital data entry using EpiCollect5 (*Oxford Big Data Institute*). Local research assistants also coded patient addresses as being “slum” or “non-slum” as a proxy for economic status.

### Definitions

We defined the treatment phase as the period between the treatment initiation month recorded at cohort entry and the database-recorded month of treatment completion unless the patient reported continuing treatment outside the PPIA program, in which case the end of the treatment phase was defined as the self-reported month of treatment cessation. We defined the post-treatment phase as the period between the end of the treatment phase and the month that the patient completed the survey. The end of the post-treatment phase is also the point of censoring from end of follow-up. Thus, the post-treatment phase duration for each patient is variable. Patients who completed treatment earlier will have had potentially more time in the post-treatment phase before being censored at the survey date than patients who finished treatment more recently. To account for this, time spent in the post-treatment phase is accounted for in all analyses. Patients could experience fatality either during the treatment or post-treatment phase (**Fig A in S1 File**). Patients could only experience recurrence during the post-treatment phase. Patients could experience recurrence before experiencing post-treatment fatality. As only patients who responded to the survey are used our IPS weighted analysis, censoring only occurred due to end of follow-up at the time of responding to the survey.

We defined the case fatality ratio, a measure of all-cause mortality, as the proportion of patients who died from any cause during the treatment or post-treatment phase divided by the number of patients starting the relevant phase. As we were limited by the nature of our phone survey, we defined the recurrence rate as the proportion of patients in the post-treatment phase who reported initiating another round of TB treatment.

Adherence proportion was defined as the average proportion of doses reported taken by the patient during biweekly adherence monitoring calls. Adherence proportion was categorized in some analyses into patients who received less than one month of doses (“<1 month Adherence”), patients who received between one month and 80% of doses (“Poor Adherence”), and patients who received more than 80% of doses (“Good Adherence”).

### Sample size calculation

Sample size was calculated to give adequate precision to the phase-specific event rates.

A 4,000 patient sample assuming a 5% CFR during treatment (based on reports from the Indian national TB program [16]) and independent observations, would give a treatment CFR with a margin of error of 0.68%. Conservatively assuming that 3,000 of these patients enter the post-treatment phase, at a 5% event rate, the post-treatment CFR and recurrence proportion would have a margin of error of 0.78%.

These margins of error were deemed sufficiently precise for the meaningful estimation of the CFRs and recurrence rate.

### Missing baseline data and imputation

The rate of missingness in the data collected at enrollment in was low: data were missing from the slum address classification (n = 259, 6.5%) and in the PPIA reported treatment phase duration (n = 21, 0.5%). When missingness is this low, the choice of imputation method is unlikely to substantially influence the results; thus, a single chained imputation [17] was performed. The raw demographics are summarized in Table 1. In all models, the imputed data were used.

**Table 1:** Summary of baseline cohort demographics, n = 4000.

	Total Cohort (n = 4000)		Observed (n = 2240)		Unobserved (n = 1760)	
	n	%	n	%	n	%
Female	1615	40.4	915	40.8	700	39.8
Mean age	30.4, SD = 18.8		30.8 SD = 19		29.9 SD = 18.5	
EPTB	1256	31.4	770	34.4	486	27.6
Retreatment	427	10.7	229	10.2	198	11.2
Resides in slum	1800	45.0	1023	45.7	777	44.1
< 1 month of treatment adherence	867	21.7	303	13.5	564	32.0
Poor adherence	1840	46.0	1016	45.4	824	46.8
Urban Patna	2023	50.6	1191	53.2	832	47.3
Rural Patna	720	18.0	335	15.0	385	21.9
Out of Patna	1257	31.4	714	31.9	543	30.9

Abbreviations: SD–standard deviation, EPTB–extrapulmonary TB

### Loss to follow up and inverse probability selection weighting

Despite best efforts to contact patients, some could not be reached. It is possible that patients who could not be surveyed experienced differential rates of fatality or recurrence, thus excluding nonresponsive patients from analyses would bias the resulting event rates. To correct for this selection bias, IPSW was applied [14]. This method takes advantage of the baseline demographic variables recorded at cohort entry. Demographic variables theorized to be related to the risk of fatality and/or recurrence and the probability of completing the survey were used to construct a model predicting the probability of competing the survey. This predicted probability was inverted to create a weight. By applying these selection weights, observed patients were re-weighted to represent themselves and unobserved patients allowing for selection bias-corrected analyses.

The following baseline variables were available in the PPIA database and were hypothesized, based on prior research [3, 18–21], to be related to both the likelihood of response and risk of fatality and/or recurrence: age, gender, PTB/EPTB, new/retreatment case, slum/non-slum address, urban/rural/out-of-Patna address, PPIA reported treatment duration, treatment adherence and date of treatment initiation.

### Sensitivity analysis: Truncated weights

Rare combinations of demographics among observed patients can result in large IPS weights creating highly influential patients. To assess the sensitivity of our results to outlier weights, the primary analysis was recalculated after truncating the weights to fall within the 1^st^ and 99^th^ percentile.

### Case fatality ratios and recurrence rates

A case fatality ratio for the entire treatment phase weighted by IPSW was estimated. Case fatality ratios and recurrence rates also weighted by IPSW were calculated at 3, 6, 9, 12, 18, and 24 months in the post-treatment phase. Corresponding unweighted CFRs and recurrence rates were also estimated. All proportion confidence intervals were empirically bootstrapped one thousand times for both the weighted and un-weighted proportions. Confidence intervals were taken as the 2.5^th^ and 97.5^th^ percentile of the resulting proportion distribution [22].

### Survival curves and survival modelling

Kaplan-Meir curves were created for treatment and post-treatment phase fatality and post-treatment phase TB recurrence, all weighted using IPSW.

Multivariable Cox proportional hazards models [23] were used to estimate adjusted hazard ratios (HRs) for fatality from any cause during the treatment and post-treatment phases weighted with IPSW. For the treatment phase fatality model, patients were followed from the month of treatment initiation until fatality or censoring due to treatment completion (the defined end of the treatment phase). In the post-treatment phase fatality model, patients were followed from the month of treatment completion to fatality or censoring at the survey date. The proportionality assumptions were verified using Schoenfeld residuals [24]. In the multivariable Cox proportional hazard models, treatment adherence was modelled flexibly using penalized splines [25, 26] with four degrees of freedom. If required to maintain proportionality, treatment phase fatality models included a time term, either linearly, as an interaction with a coefficient or using penalized splines (df = 4).

As death is a competing risk for recurrence which can bias standard Cox proportional hazards models, a Fine and Gray sub-distributional hazard model [27] was estimated for post-treatment phase recurrence weighted with IPSW. In this model, patients were followed from the month of treatment completion to either 1) recurrence, 2) the competing event, fatality, or 3) censoring at the survey date. Adherence was modelled categorically (“<1 month adherence”, “poor adherence”, “good adherence”, see Definitions) as the introduction of splines created convergence issues.

All models used month since the beginning to the treatment or post-treatment phase as the time scale. Models were adjusted for gender, age, PTB/EPTB, new/retreatment case, region (Patna urban/Patna rural/out of Patna), slum/non-slum address and treatment adherence. Post-treatment models were additionally adjusted for duration of treatment. Data on measures of disease severity or comorbid conditions were not available.

Unweighted models of identical forms are also presented for comparison.

All coefficient confidence intervals for all survival models were empirically bootstrapped one thousand times. Confidence intervals were taken as the 2.5^th^ and 97.5^th^ percentile of the resulting coefficient distribution [28].

### Ethics

World Health Partners, a TB service delivery program, collected the patient follow-up data as part of their ongoing PPIA management and assessment with approval from local and national tuberculosis program authorities. As this survey was part of the PPIA program’s internal performance assessment, it is categorized as operational research and an ethics board approval was not sought for the survey. Approval for secondary data analysis by the McGill co-authors of the survey data was obtained from McGill University (A02-M05-18B).

### Patient and public involvement

This research study was designed without patient or public involvement.

## Results

**Fig 1:**
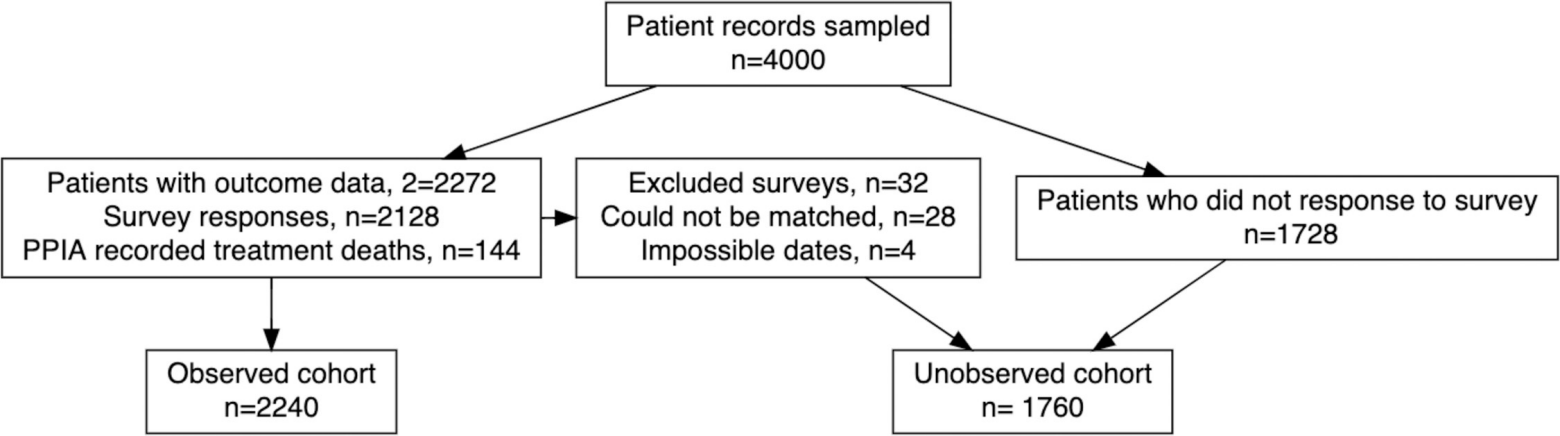
Flow chart of patient sampling and surveying.

Of the 4,000 patients in the sample, 2,128 (2,128/4000, 53.2%) patients were contacted and consented to the survey. Most patients were reached by phone (n = 2029, 95.3%) but 99 patients (4.7%) were only reached by home visit. Because of linkage errors (n = 28) or impossible dates (n = 4), 32 (32/2128, 1.5%) patient responses were discarded. Including deaths recorded in the PPIA database (n = 144), 2,240 (2240/4000, 56.0%) of patients had complete records and form the observed cohort (Fig 1).

### Cohort characteristics

A summary of the baseline characteristics of the total 4,000 patient sample as well as the observed and unobserved patient subsets is presented in Table 1. The average age in the total patient sample was 30.4 years and 40.4% of patients were female. A third of patients had EPTB (n = 1,256, 31.4%) and 10.7% were retreatment cases. Almost half of the cohort resided in a slum (n = 1,800, 45.0%). Patient reported treatment adherence was low with 21.7% reporting less than a month of treatment adherence to the call center, and 46.0% reporting less than 80% of doses. Half of the cohort lived in urban Patna (n = 2,023, 50.6%), 18.0% lived in rural Patna, and 31.4% resided outside of Patna city limits.

In total, 2,240 patients were included in the observed cohort (Fig 1). Unobserved patients were less likely to have EPTB compared to observed patients (27.6% vs. 34.4%), suggesting that unobserved patients may have experienced more severe disease. Unobserved patients were more likely to report less than one month of treatment adherence (32.0% vs. 13.5%) and more likely to live in rural Patna (21.9% vs. 15.0%).

### Inverse probability selection weights

The IPS weights from the selection model (**Equation A in S1 File**) had a median of 1.49 and ranged from 1.05 to 8.61. The 10^th^ and 90^th^ percentiles were 1.27 and 2.54 respectively, meaning there were very few highly influential patient weights (**Fig B in S1 File**). The weights produced excellent covariate balance between the observed and full cohort (Table 2).

**Table 2:** Covariate balance between full cohort and the IPS weighted observed cohort.

	Full cohort		IPS weighted observed cohort	
	Mean	Variance	Mean	Variance
Age	30.38	352.76	30.47	353.73
% Female	0.40	0.24	0.40	0.24
% PTB	0.69	0.22	0.68	0.22
% New	0.89	0.10	0.89	0.10
% Patna Urban	0.51	0.25	0.51	0.25
% Out of Patna	0.31	0.22	0.30	0.21
% Slum, imputed	0.48	0.25	0.48	0.25
Treatment months (PPIA reported), imputed	7.61	17.03	7.39	15.72
Average % Adherence	0.51	0.14	0.53	0.13

#### Treatment phase case fatality

The weighted average patient-reported treatment duration was 8.7 months (8.6 months unweighted average).

#### Case fatality ratio

The unweighted treatment phase CFR was 4.15% (3.56%, 4.83%) and the weighted treatment phase CFR was 7.27% (5.97%, 8.49%).

#### Survival curve and modelling

**Fig 2:**
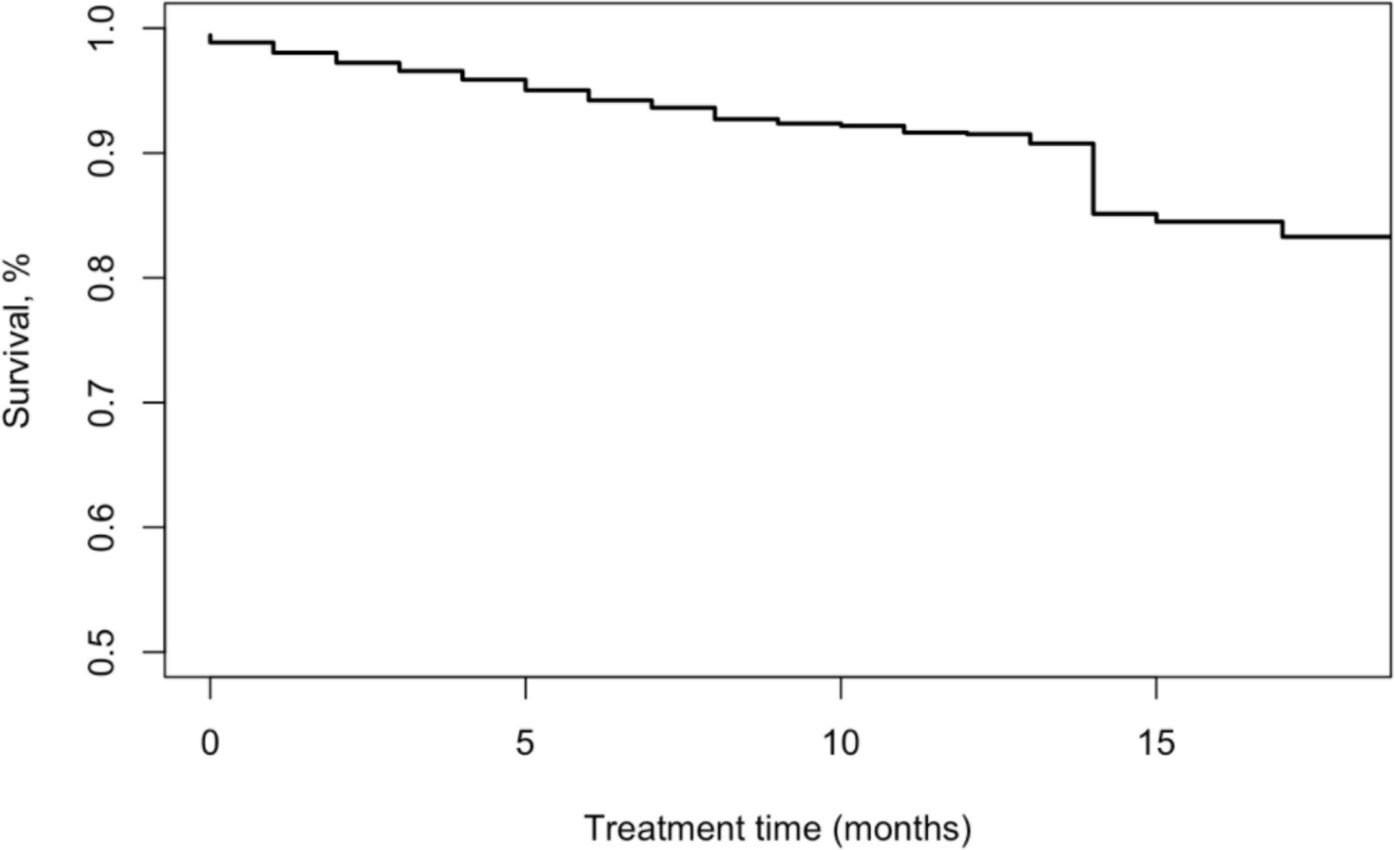
Treatment phase Kaplan-Meir survival curve weighted using IPSW. Follow-up period begins at treatment initiation and continues until fatality or censoring by self-reported treatment completion. Treatment time refers to the months of treatment reported by the patient.

**Fig 3:**
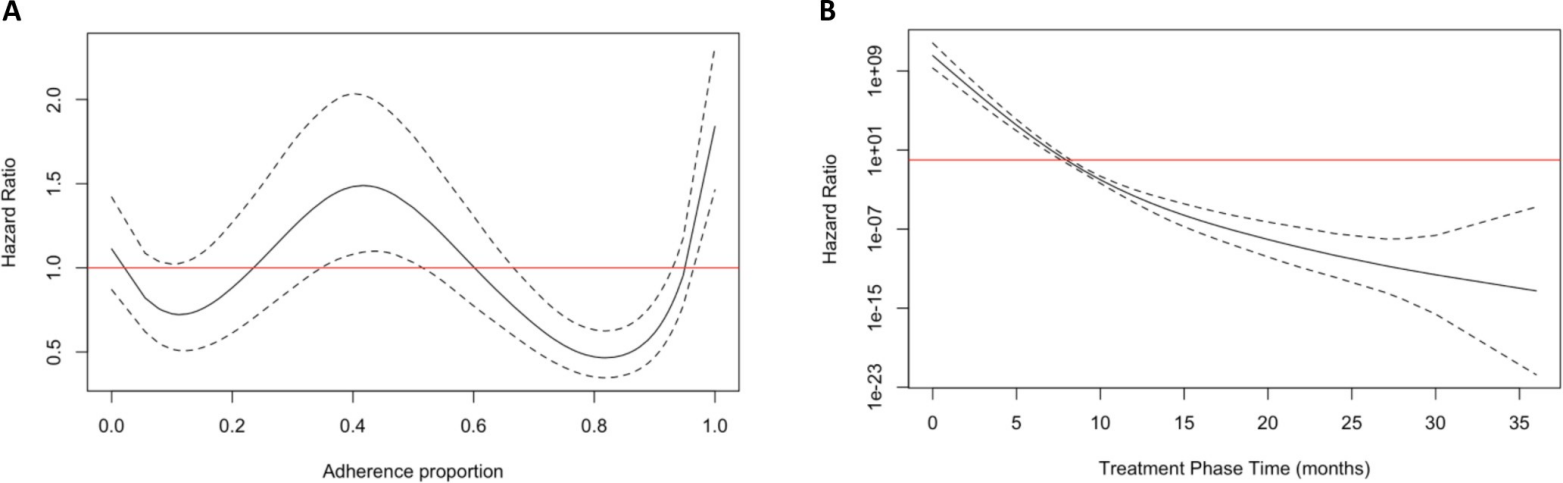
Penalized spline functions (df = 4) from the treatment phase fatality model. (A) The solid black line is the estimated hazard ratio (y-axis) for adherence proportion (x-axis) with all other variables in the model held constant. (B) The solid black line is the estimated log hazard ratio (y-axis) for treatment phase time in months (x-axis) with all other variables in the model held constant. Dashed black lines are the non-bootstrapped confidence intervals. Red lines indicate the null HR of one.

Case fatality occurred fairly linearly through the treatment phase (Fig 2). Some patients had treatment phases that outlasted the standard 6–9 month treatment duration for drug-sensitive TB as some private providers advised patients to continue treatment past what was provided for free by the PPIA. Older patients were significantly more likely to die with a hazard ratio (HR) of 1.03 (1.02, 1.04) for each additional year in age (Table 3). Adherence was associated with a higher hazard of fatality around 40% adherence and a lower hazard around 80% adherence (Fig 3A). Paradoxically, near perfect adherence was associated with increased hazard; this may be driven by confounding by indication, where the sickest patients were more likely to be highly adherent to treatment or given more support by the care providers. The risk of fatality decreased substantially with time (Fig 3B).

**Table 3:** Weighted and unweighted treatment phase case fatality Cox proportional hazards model hazard ratios.

	Unweighted model hazard ratio (95% CI)	Weighted model hazard ratio (95% CI)
Male	Ref	Ref
Female	0.75 (0.54, 1.02)	0.71 (0.47, 1.05)
Age (per year)	**1.03 (1.02, 1.03)**	**1.03 (1.02, 1.04)**
New	Ref	Ref
Retreatment	1.37 (0.86, 2.14)	1.34 (0.74, 2.26)
Out of Patna	Ref	Ref
Patna Rural	0.90 (0.57, 1.33)	0.99 (0.58, 1.56)
Patna Urban	0.72 (0.51, 1.08)	0.79 (0.53, 1.15)
PTB	Ref	Ref
EPTB	0.95 (0.68, 1.31)	0.84 (0.57, 1.19)
Non-slum	Ref	Ref
Slum	0.81 (0.60, 1.07)	0.72 (0.51, 1.00)

### Post-treatment phase outcomes

A total of 2,078 surveyed patients entered the post-treatment phase; after weighting they represent 3,642 patients. The weighted average post-treatment phase duration was 26.4 months (25.8 months unweighted average). A histogram of the crude post-treatment follow-up times is available in **(Fig C in S1 File)**; 53.5% of crude post-treatment follow-up times exceeded two years.

#### Case fatality ratio

Unweighted and weighted post-treatment CFRs are available in Table 4. At 24 months into the post-treatment phase, the unweighted post-treatment phase CFR was 3.53% (2.59%, 4.78%) and the weighted post-treatment phase CFR was 3.32% (2.36%, 4.42%).

**Table 4:** Unweighted and weighted post-treatment CFRs.

Months post-treatment	Unweighted Post-treatment CFR % (95% CI)	Weighted Post-Treatment CFR % (95% CI)
3	0.44 (0.21, 0.86)	0.39 (0.15, 0.65)
6	0.83 (0.50, 1.35)	0.76 (0.42, 1.18)
9	1.04 (0.66, 1.62)	0.92 (0.54, 1.35)
12	1.34 (0.90, 1.99)	1.23 (0.75, 1.73)
18	2.16 (1.53, 3.03)	2.03 (1.39, 2.69)
24	3.53 (2.59, 4.78)	3.32 (2.36, 4.42)

#### Survival model

Post-treatment phase case fatality occurred fairly linearly throughout the post-treatment phase (**Fig D in S1 File**).

**Fig 4:**
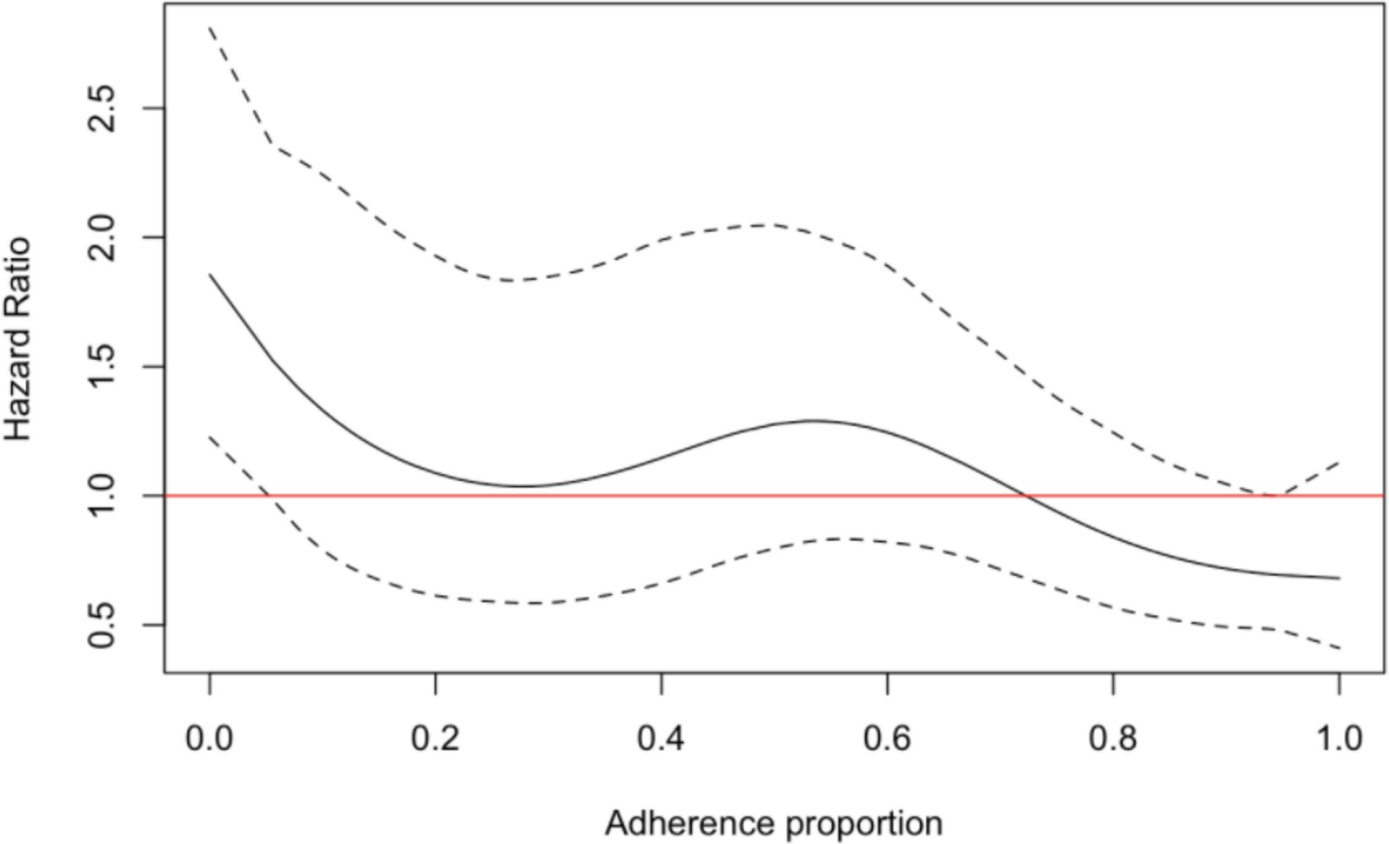
Penalized spline functions (df = 4) from the post- treatment phase fatality model. The solid black line is the estimated hazard ratio (y-axis) for adherence proportion (x-axis) with all other variables in the model held constant. Dashed black lines are the non-bootstrapped confidence intervals. Red lines indicate the null HR of one.

Older patients were more likely to die during the post-treatment phase with a HR of 1.06 (1.05, 1.08) per year of age (Table 5). Patients living in rural Patna were less likely to die compared to patients who lived outside city bounds (HR: 0.39, [0.11, 0.88]). The risk of fatality during the post-treatment phase trended downwards with increasing treatment adherence, but the only significant increase in hazard occurred at extremely low adherence (Fig 4).

**Table 5:** Unweighted and weighted post-treatment phase case fatality Cox proportional hazards model hazard ratios.

	Unweighted model hazard ratio (95% CI)	Weighted model hazard ratio (95% CI)
Male	Ref	Ref
Female	0.96 (0.49, 1.74)	0.96 (0.52, 1.79)
Age (per year)	**1.06 (1.05, 1.07)**	**1.06 (1.05, 1.08)**
New	Ref	Ref
Retreatment	1.38 (0.39, 3.07)	1.70 (0.51, 3.68)
Out of Patna	Ref	Ref
Patna Rural	**0.39 (0.10, 0.86)**	**0.39 (0.11, 0.88)**
Patna Urban	0.60 (0.33, 1.10)	0.61 (0.32, 1.14)
PTB	Ref	Ref
EPTB	0.85 (0.35, 1.61)	0.97 (0.44, 1.88)
Non-slum	Ref	Ref
Slum	1.45 (0.82, 2/73)	1.29 (0.69, 2.50)
Months of treatment	1.00 (0.92, 1.07)	0.98 (0.90, 1.05)

#### Recurrence rate

Unweighted and weighted post-treatment recurrence rates are available in Table 6. At 24 months into the post-treatment phase, the unweighted post-treatment phase recurrence rate was 3.73% (2.77%, 5.01%) and the weighted post-treatment phase recurrence rate was 3.56% (2.54%, 4.79%).

**Table 6:** Unweighted and weighted post-treatment recurrence rates.

Months post-treatment	Unweighted Post-treatment recurrence rate % (95% CI)	Weighted Post-Treatment recurrence rate % (95% CI)
3	0.29 (0.12, 0.66)	0.29 (0.08, 0.55)
6	0.59 (0.32, 1.05)	0.56 (0.23, 0.91)
9	0.74 (0.43, 1.25)	0.73 (0.36, 1.14)
12	1.19 (0.77, 1.81)	1.23 (0.72, 1.80)
18	1.96 (1.37, 2.79)	1.89 (1.27, 2.62)
24	3.73 (2.77, 5.01)	3.56 (2.54, 4.79)

#### Recurrence-free survival model

The risk of a recurrent episode of TB occurred linearly throughout the post-treatment phase (**Fig E in S1 File**). No significant associations with the risk of recurrence in the presence of death as a competing risk were found in the weighted sub-distributional hazard models (Table 7).

**Table 7:** Post-treatment phase recurrence Fine and Gray survival model sub-distribution hazard ratios.

	Unweighted sub-distribution Hazard Ratio (95% CI)	Weighted sub-distribution Hazard Ratio (95% CI)
Male	Ref	Ref
Female	1.02 (0.63, 1.59)	1.03 (0.61, 1.67)
Age (per year)	0.99 (0.98, 1.01)	1.00 (0.99, 1.01)
New	Ref	Ref
Retreatment	1.41 (0.61, 2.54)	1.27 (0.54, 2.57)
Out of Patna	Ref	Ref
Patna Rural	1.97 (0.93, 3.83)	1.82 (0.86, 3.81)
Patna Urban	0.96 (0.62, 2.02)	0.91 (0.55, 1.57)
PTB	Ref	Ref
EPTB	0.96 (0.56, 1.68)	1.01 (0.58, 1.63)
Non-slum	Ref	Ref
Slum	0.71 (0.45, 1.10)	0.68 (0.40, 1.14)
Good Adherence (>80% of doses)	Ref	Ref
< 1 month of treatment adherence	0.86 (0.33, 1.63)	0.71 (0.23, 1.42)
Poor Adherence (<80% of doses)	1.14 (0.72, 1.89)	1.07 (0.64, 1.80)
Months of treatment	1.05 (0.97, 1.09)	1.05 (0.97, 1.10)

### Truncated weights sensitivity analysis

The 1st and 99th percentile IPW weights were 1.10 and 5.19, respectively. As a sensitivity analysis, the primary outcomes were recalculated with the weights truncated to within this range (**Table A in S1 File**). The results after truncating the weights are nearly identical indicating that this analysis is not sensitive to outlier IPS weights.

## Discussion

The estimated IPS weighted average patient reported treatment duration was 8.7 months as many patients continued treatment with their physician after using the 6 months provided for free by the PPIAs and the weighted treatment phase CFR was 7.27% (5.97%, 8.49%), which is higher than the target CFR called for by the WHO End TB Strategy [2]. The adjusted CFR is nearly two-fold higher than crude unweighted CFR (4.15% [3.56%, 4.83%]) suggesting that patient loss to follow-up severely biased the crude estimates. Patients lost to follow-up were much more likely to have minimal treatment adherence, a major risk factor for poor outcomes. Treatment phase fatality was predicted by age (HR: 1.03 [1.02, 1.04]) and was associated low treatment adherence (Fig 3A).

Our study benefited from a long post-treatment follow-up period; the average weighted post-treatment phase duration was 26.4 months. The IPS weighted CFR at 24 months into the post-treatment phase was 3.32% (2.36%, 4.42%). The difference between the weighted and unweighted post-treatment phase CFRs was less than that observed in the treatment phase CFRs. This suggests that loss to follow-up was related to risk of death during the treatment phase but that missingness in the post-treatment phase was closer to missingness at random.

Post-treatment fatality was associated with age (HR: 1.06 [1.05, 1.08]) and poor adherence (Fig 4). Living in rural Patna was associated with a lower hazard of post-treatment fatality (HR: 0.39 [0.11, 0.88]).

Very few studies have investigated post-treatment recurrence rates but previous literature estimates were as high as 10% [12]; our study found a IPS weighted recurrence rate of 3.56% (2.54%, 4.79%) 24 months into the post-treatment phase. The relatively low recurrence rate observed here suggests that the PPIA treatment regimen was effective. However, any number of patients suffering a second bout of TB highlights the failure to correct socioeconomic conditions that leave patients vulnerable to TB infection. Recurrence occurred evenly throughout the post-treatment period (**Fig D in S1 File**), suggesting that most recurrences were not a relapse, which we would expect early in the post-treatment phase.

### Strengths and limitations

This work represents the largest cohort study of outcomes among privately treated Indian TB patients to date; a group historically difficult to study because of the fragmented nature of the private healthcare sector. Our study benefited from a long period of post-treatment phase follow-up but this comes at a cost to response rate. Our response rate (56%) was likely impacted by both seasonal economic migration of our study population and frequent phone numbers changes common among Indians in response to phone carrier incentives. However, this work benefits from the application of IPSW to adjust for potential bias due to loss to follow-up, a novel application in this area. An additional important limitation is that we could not assess cause of death through our phone surveys, meaning that our CFRs reflect all-cause mortality. The WHO defines any death that occurs during TB treatment as a TB-related death [1] but in future work it would be useful to investigate cause-specific death rates in the post-treatment phase. If patient deaths were investigated prospectively, verbal autopsy [29] could be used to assign cause of death, as many Indians die at home and do not receive death certificates [30]. Other studies have suggested that TB patients are more susceptible to cardiovascular disease after treatment completion than the general population [31]. Another limitation to this work is that PPIAs likely represent the best-case scenario for treatment quality in India’s largely unregulated private healthcare sector. Many patients in the private sector do not receive the standard WHO TB treatment regimen [5] or may receive substandard drugs [32], thus the PPIA patient experience is not universal. Additionally, it is possible that some patients were treated by the same physician and that these clustered patients may have correlated data. We did not account for this clustering because provider codes were not provided in the de-identified data used for these analyses. We do not anticipate this potential clustering to meaningfully impact results as regardless of provider, each patient received the same standard drug regimen and treatment adherence monitoring. If the intra-cluster correlation within provider group was substantial, our estimated standard errors may be smaller than the true standard errors. Our study was limited to patient self-report for both treatment adherence and recurrence of TB. Patients may have felt pressure to report higher than actual treatment adherence to the call center counsellor. The ideal measure of recurrent TB would involve symptom screening and TB testing, however because of our design we were restricted to asking if patients had initiated another round of TB treatment. This definition means we may have missed patients who had begun to show TB symptoms but had not yet been diagnosed. We may also have included patients who had received medication for a disease other than TB, but erroneously reported this as a recurrent TB episode. Data on TB disease severity or comorbid conditions were not collected in the PPIA databases, thus the influence of these factors on fatality and recurrence could not be assessed. Similarly, as no economic information was collected at PPIA enrollment, our only means to measure household income was classifying the patient’s address as slum/non-slum. This is an imperfect measure of economic status as income within and outside slums can vary substantially. We recommend future PPIAs and similar programs collect household income information at enrollment to facilitate future analyses.

Finally, in order for IPSW to provide unbiased weighted estimates, the probability of selection model must contain all variables that are related to the probability of response and the probability of experiencing the outcome events. There are variables known to influence the risk of TB fatality–like smoking [33], HIV status [34] and malnutrition [35]–that were not available in the PPIA database. Future simulation work is planned to investigate the robustness of these results to these unmeasured variables. Additionally, other selection bias correction methods are available and may have produced different results than IPSW. Multiple imputation is often more efficient than IPSW [14], but IPSW allows for an examination of the relationships between patient characteristics and non-response.

### Implications

PPIAs are an increasingly common response to ensuring even and high-quality TB care in the large Indian private healthcare sector. In this Patna-based PPIA cohort, we observed near ideal treatment phase CFRs and moderately low rates of post-treatment phase fatality and recurrence. This favorable evaluation supports the continued roll-out of PPIAs to improve private sector TB care. However, PPIAs would benefit from incorporating systematic routine quality assessment and cascade of care analyses [36]. Given that PPIAs already use call centers to provide adherence counselling, it would be straightforward to include post-treatment monitoring for long-term outcomes. Patient outcomes both during and after treatment could be monitored and reported through “dashboards” that could highlight where improvements could be made in the cascade of care.

Methodologically rigorous studies of Indian TB patient outcomes are too rare, indeed this work is the first in the literature to apply a correction for selection bias induced by loss to follow-up [3]. This correction substantially elevated the estimate for treatment phase case fatality, demonstrating that TB patient cohort studies, like most cohort studies, are susceptible to selection bias. To make accurate evaluations of TB program effectiveness, researchers and TB programs alike must account for missing data. To make the best decisions to improve patient quality of care, we must first accurately measure the current state of affairs.

Finally, this work highlights that TB patients are still at risk after their treatment ends. Around 3% of patients had died only two years after treatment; almost 4% had started another round of TB treatment. These fatality and recurrence rates could be reduced with continuing contact with the healthcare system and efforts to improve the socioeconomic status of TB patients. Future work will include relative survival analysis comparing post-treatment TB patient fatality rates to the general population rates and verbal autopsy studies to assess causes of death.

## Supporting information

S1 File (PDF)
