# Supplementary material for: Case fatality and recurrent tuberculosis among patients managed in the private sector: A cohort study in Patna, India
Source: PLoS One. 2021 Mar 26;16(3):e0249225. doi: 10.1371/journal.pone.0249225 (PMC7996982; doi:10.1371/journal.pone.0249225)
Supplement: S1 File — (PDF) [file pone.0249225.s001.pdf]

Tuberculosis case fatality and recurrence in a private sector cohort in Patna, India

Sophie Huddart[1,2,3], Mugdha Singh[4], Nita Jha[4], Andrea Benedetti[1], Madhukar Pai[1,2,5]

Affiliations

1. Department of Epidemiology, Biostatistics and Occupational Health, McGill University, Montreal, Canada
2. McGill International TB Centre, Montreal, Canada
3. Faculty of Medicine, University of California – San Francisco, San Francisco, USA
4. World Health Partners, Patna, India
5. Manipal McGill Centre for Infectious Diseases, Manipal Academy of Higher Education, Manipal, India

ONLINE SUPPLEMENTAL MATERIALS

Methods

Definitions

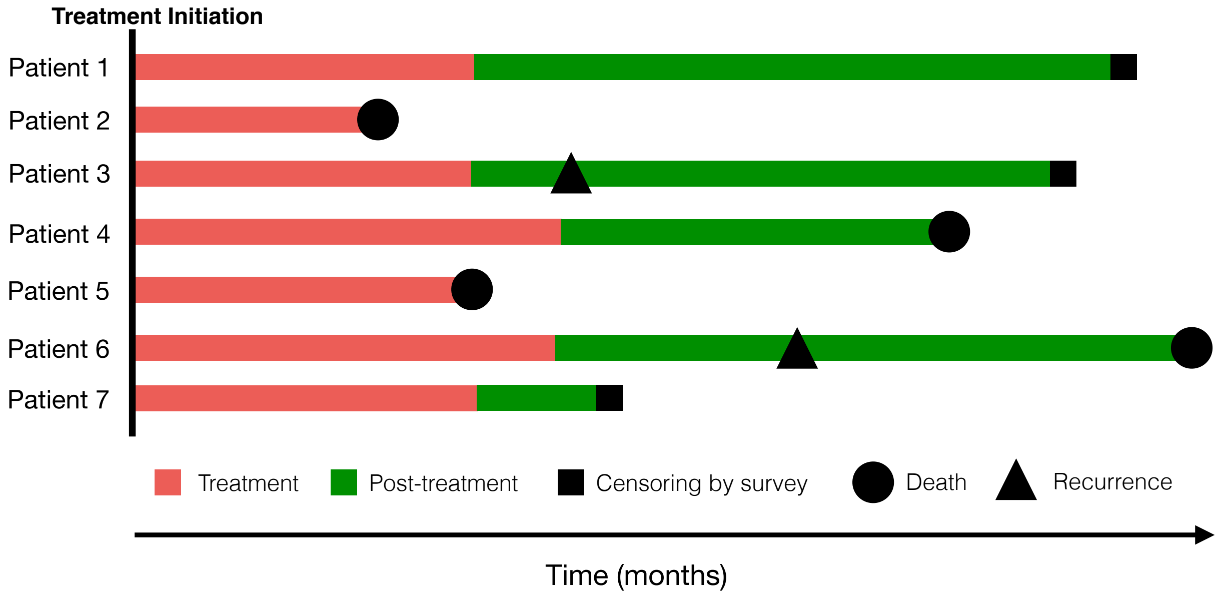

Figure A, Diagram of hypothetical patient follow-up periods. Red indicates the treatment phase, which is defined from treatment initiation to PPIA-reported or patient-reported treatment cessation, whichever is longer. Green indicates the post-treatment phase, which is defined from treatment cessation to the date of survey or fatality or recurrence.

Results

$$S \sim gender_{imp} + spline(age) + TB\ site + Case\ type + slum_{imp} + spline\left(adherence_{imp}\right) + spline(time\ since\ initiation) + spline(PPIA\ treatment\ duration) + region$$

where S is observation status, "imp" indicates imputed, TB site refers to PTB/EPTB, case type refers to new/retreatment cases, time since initiation was the number of calendar months since the patient initiated treatment and June 2019, PPIA treatment duration is the number of months of treatment recorded in the PPIA database and region refers to out of Patna/Patna urban/Patna rural . All categorical variables were modelled using dummy variables. Splines were penalized splines with df=4

Equation A, IPSW selection model

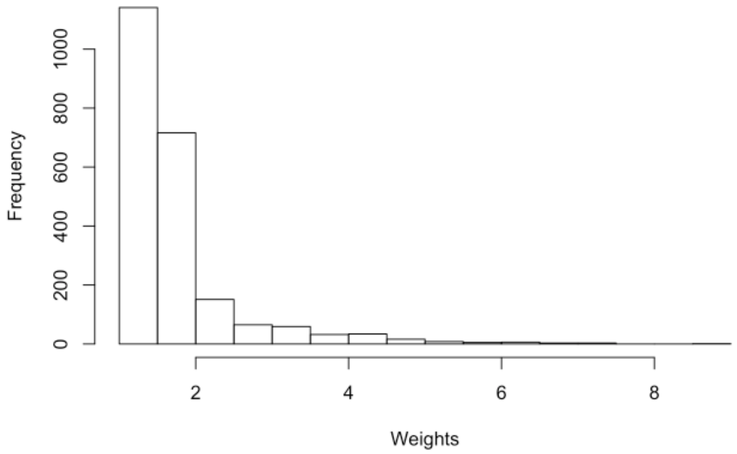

Figure B, Histogram of IPS weight distribution

<u>Histogram of post-treatment crude follow-up times</u>

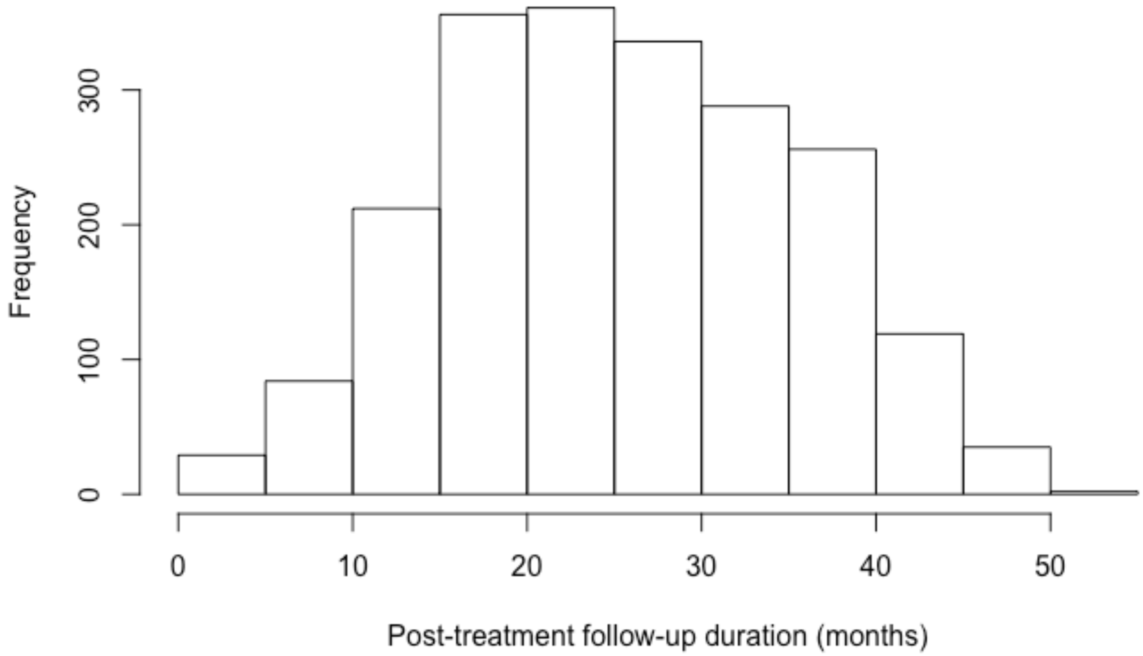

Figure C, Histogram of post-treatment crude follow-up times.

<u>Post-treatment phase survival curve</u>

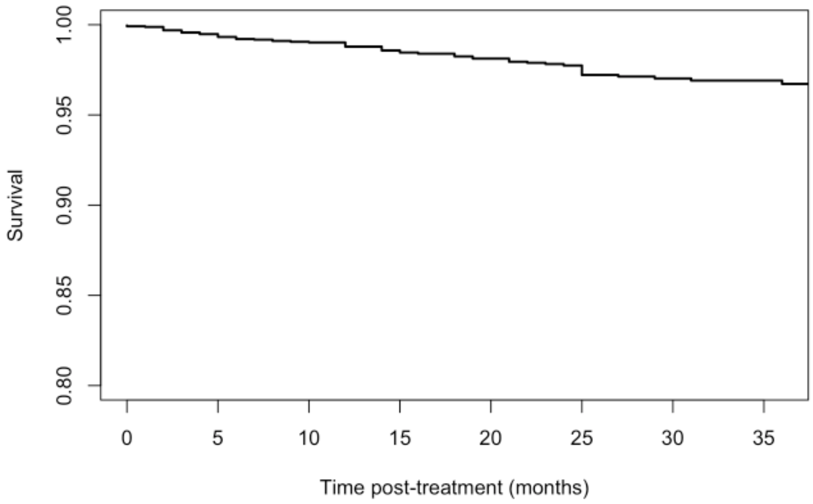

Figure D, Post-treatment phase Kaplan-Meir survival curve weighted using IPSW. Follow-up period begins at treatment cessation and continues until fatality or censoring by survey date.

Post-treatment phase recurrence-free survival curve

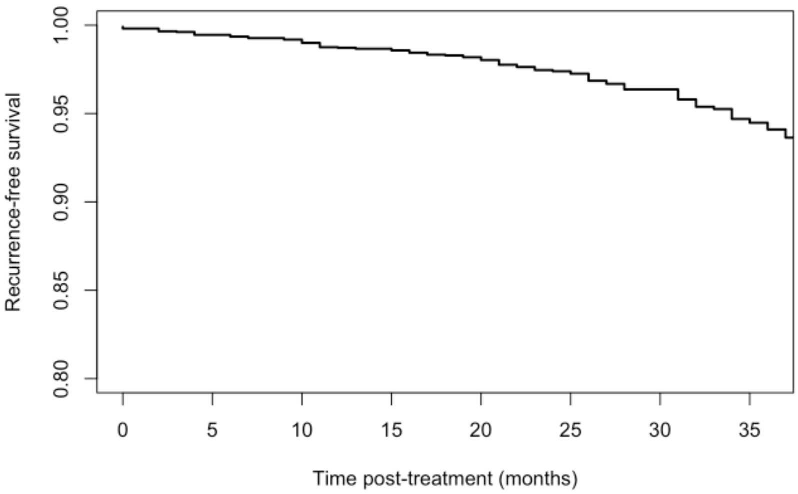

Figure E, Post-treatment phase Kaplan-Meir recurrence-free survival curve weighted using IPSW. Follow-up period begins at treatment cessation and continues until recurrence or censoring by fatality or survey date.

Table A, Comparison of primary analysis and truncated weight sensitivity analysis

**Treatment Phase**		**Post-treatment Phase**				
**Full weights CFR % (95% CI)**	**Truncated weights CFR % (95% CI)**	**Months post-treatment**	**Full weights CFR % (95% CI)**	**Truncated weights CFR % (95% CI)**	**Full weights recurrence rate % (95% CI)**	**Truncated weights recurrence rate % (95% CI)**
7.27 (5.97, 8.49)	7.18 (6.90, 8.40)	3	0.39 (0.15, 0.65)	0.39 (0.16, 0.67)	0.29 (0.08, 0.55)	0.29 (0.07, 0.55)
		6	0.76 (0.42, 1.18)	0.76 (0.42, 1.13)	0.56 (0.23, 0.91)	0.56 (0.26, 0.92)
		9	0.92 (0.54, 1.35)	0.93 (0.52, 1.38)	0.73 (0.36, 1.14)	0.74 (0.38, 1.16)
		12	1.23 (0.75, 1.73)	1.23 (0.75, 1.74)	1.23 (0.72, 1.80)	1.23 (0.71, 1.80)
		18	2.03 (1.39, 2.69)	2.04 (1.4, 2.77)	1.89 (1.27, 2.62)	1.90 (1.20, 2.66)
		24	3.32 (2.36, 4.42)	3.34 (2.38, 4.44)	3.56 (2.54, 4.79)	3.58 (2.52, 4.64)
